# Dentition Anomalies and Cranial Abnormalities in Invasive Beavers (*Castor canadensis*) in Tierra Del Fuego, Argentina

**DOI:** 10.3390/ani14162285

**Published:** 2024-08-06

**Authors:** Alvaro González-Calderón

**Affiliations:** Instituto de Ecología, Genética y Evolución de Buenos Aires (UBA-CONICET), Departamento de Ecología, Genética y Evolución, Facultad de Ciencias Exactas y Naturales, Universidad de Buenos Aires, Intendente Güiraldes 2160, Buenos Aires 1428, Argentina; alvaroglez3@gmail.com

**Keywords:** hyperdontia, hypodontia, invasive mammal, caries, malocclusion

## Abstract

**Simple Summary:**

The dentition analysis in vertebrates has been a pillar for the development of the classic and alternative theories on the evolution of species. The early dentition pattern consists of numerous, similar, and simple conical teeth, which are typically repeated in each half of both the maxilla and mandible. In mammals, however, dentition is regionally differentiated according to the dental arch, with dietary specialization being the main evolutionary pressure that defines the number, size, and shape of teeth. The developmental failure of a tooth normally present (hypodontia) and duplication of teeth (hyperdontia) is a deviation from the typical dental formula; their analysis allows for determining the prevalence of some dental conditions and their role in the health status of populations. After examining a sample size of 970 skulls of a total of 1121 beavers (*Castor canadensis*) that were removed in invaded areas of Tierra del Fuego, two dentition anomalies were recorded: hypodontia and hyperdontia (0.41% in both cases). Beavers also presented artifactual tooth loss (0.30%), acquired tooth loss (0.61%), fractured teeth (0.41%), and caries (0.31%). These conditions occurred in all age classes, showing a female bias, and represent a precedent of dentition anomalies and dentition and cranial conditions in an exotic invasive mammal.

**Abstract:**

The study of dentition anomalies and pathologies in wildlife contributes, together with other indicators, to understanding the relevance of some factors on the health status of populations. This has not been properly evaluated in invasive mammals. To test the feasibility of eradication, the government of Tierra del Fuego performed the eradication of beavers (*Castor canadensis*) from 2016 to 2018: 1121 animals were removed and 970 examined. These beavers were examined to analyze the incidence of dentition anomalies and other dentition and cranial conditions. The beavers presented two dentition anomalies: hypodontia and hyperdontia (0.41% in both cases), and for the first time, a dentition anomaly in the upper quadrants was reported. Beavers also presented artifactual tooth loss (0.30%) and acquired tooth loss (0.61%) but with low incidence. The presence of fractured teeth (0.41%) and caries (0.31%) occurred in all age classes, also with low incidence. The third molar tooth was the most affected, showing a female bias. The 0.93% of skulls had a cranial abnormality represented as a buccal curvature. Malocclusion cases also were recorded (0.51%). The low prevalence of dentition anomalies, fractured teeth, caries, and cranial abnormalities would not compromise the lifespan of beavers. These results leave a precedent of dentition anomalies and dentition and cranial conditions in an invasive mammal.

## 1. Introduction

The dentition analysis in mammals—by assessment of the structure, growth patterns, and replacement of teeth—has allowed for the sex identification, age estimation, and reconstruction of life histories [1,2]. The increase and the high prevalence of dental disease (such as caries, an infection found mostly in carbohydrates based diets [3,4]), fractured teeth by physical trauma, and dentition anomalies (e.g., tooth agenesis and duplication of a tooth) in non-human mammals, have acquired a growing relevance in ecological studies [5,6,7,8,9,10,11,12,13,14,15].

The dentition anomalies can be differentiated in five classes [16]: (i) modified morphology of one or more teeth (e.g., abnormal third molar tooth composed of only the two anterior columns in an individual of *Odocoileus virginianus*, Zimmermann 1780, where the third column of the crown is missing [17]); (ii) presence of a tooth suppressed during the evolution of the group (e.g., the suppressed upper canines teeth in deer that occasionally appear in *Capreolus capreolus,* Linnaeus 1758 [18]); (iii) tooth agenesis of a tooth normally present (e.g., non-traumatic absence of lower first premolar tooth in some individuals of *Sus scrofa* [19]); (iv) duplication of a tooth (e.g., cases of hyperdontia or supernumerary teeth in *Necromys lasiurus,* Lund 1840 [12]); and (v) changes in the position of a tooth and dental rotation (e.g., dental rotation in some individuals of *Cervus elaphus*, Linnaeus 1758 [16]). Understanding the prevalence of dentition anomalies and dental diseases in wild mammal populations and their variation under various environmental pressures can provide information, together with other indicators (such as body condition and parasitic load [20]), on the relative influence of some factors in the health status of the populations, as well as adaptive processes, genetic transfer of phenotypic traits, and evolutionary processes could even be inferred.

In particular, the tooth agenesis—i.e., developmental failure of a tooth normally present [21]—(hypodontias hereafter) and duplication of teeth—presumably by an effect of the additional creation and development of a tooth germ [22]—(hyperdontia hereafter) has been described as the deviation of the typical dental formula of mammal species [11]. These non-sex-specific anomalies have been associated with odontogenic capacity, limited gene flow, and congenital character, as well as environmental and nutritional factors, although accurately assessing the causes still represents a challenge [9,14,15,16,22,23,24,25,26]. The identification of the factors that determine these anomalies may be less evident in some cases, for example, in studies with samples obtained from different sources [23]. The prevalence of hyperdontia and hypodontia has been cataloged as rare [13] to regular [5], according to the proportion of the samples inspected or the collected species, and has been reported in most mammal orders [19].

On the other hand, unlike hypodontia, the absence of one or more teeth normally present also may occur by traumatic factors (artifactual tooth loss hereafter) and by some dental diseases, such as caries, during the life of the animal (acquired tooth loss hereafter). Some of these anomalies eventually may produce malocclusion (misalignment between the teeth of the upper and lower dental arches that produce improper tooth wear [27,28]). Nevertheless, due to the logistical aspects and the high costs of capturing a representative sample of the wild population, the incidence of hypodontia, hyperdontia, caries, fractured teeth by physical trauma (fractured teeth hereafter), artifactual tooth loss, and acquired tooth loss, as well as cranial abnormalities, has not yet been properly evaluated in populations of medium-sized invasive mammals (e.g., *Ondatra zibethicus,* Linnaeus 1766 [29]), where the ecosystems occupied are not always optimal habitats for the species. However, the eradication and collection of individuals in an invasive population, such as the removed beavers in the Isla Grande de Tierra del Fuego (Tierra del Fuego, hereafter) [30], may provide basic indirect information for assessing the prevalence of these anomalies.

The beaver (*Castor canadensis*, Kuhl 1820) is the largest semi-aquatic rodent in North America. Its dentition is monophyodont (except its premolar teeth, which are diphyodont), has hypselodont incisor teeth, and its premolar teeth and molar teeth are hypsodont with an intricate lophodont structure [31]. Beaver was introduced in 1946 in Tierra del Fuego, gradually invading and impacting the Fuegian environments and wildlife [32,33]. To assess the feasibility of eradication at the local scale, the provincial government carried out, from 2016 to 2018, the eradication in seven watersheds (areas hereafter) distributed in different environments [34]. As a result of the eradication, a total of 1121 beavers were removed, and the heads of animals were collected [30]. Full details on the selection of beaver eradication areas are available in previous studies [30]. Despite the fact that there are backgrounds on hypodontia in beavers [35,36,37,38], the collected beavers in Tierra del Fuego represent a unique occasion to opportunistically analyze the incidence of dentition anomalies (hypodontia and hyperdontia) pathologies (caries, fractured teeth, artifactual tooth loss, and acquired tooth loss), and cranial abnormalities in an introduced population.

The objectives of this study were (i) to assess the prevalence of dentition anomalies (represented here by hypodontia and hyperdontia); (ii) to record the incidence of the absence of one or more teeth by traumatic factors or by caries; and (iii) to quantify the cases of fractured teeth, caries, and cranial abnormalities in beavers that were totally removed with the purpose of testing the feasibility of eradication from seven areas in Tierra del Fuego. This research aimed to answer the following questions: (1) Does the incidence of dentition anomalies, absence of teeth (by trauma or by caries), fractured teeth, caries, and cranial abnormalities vary across different invaded environments? (2) Are dentition anomalies and pathologies more common in a particular quadrant of dentition (left and right quadrants)? (3) Are dentition anomalies, pathologies, and cranial conditions equally represented in both sexes? (4) Does the incidence of dentition anomalies, absence of teeth (by trauma or by caries), fractured teeth, or caries tend to be less represented as the age class increases? and (5) What cranial abnormalities occur in invasive beavers?

Since dentition anomalies, absence of teeth (by trauma or by caries), fractured teeth, and caries can occur in both males and females, and the sex ratio at birth in beavers is 1:1 male/female [39], it is expected that these anomalies are equally represented in both sexes. In addition, as the younger animals are proportionally more numerous in the population than adults [40], it is expected a higher number of young beavers with dentition anomalies and with pathologies with reference to the total number of specimens with dentition anomalies and pathologies regarding their age class. Conversely, it is expected a lower number of adult beavers with dentition anomalies and pathologies in relation to the total number of animals with dentition anomalies and pathologies. This study acknowledges that a representative sample of beavers in their native range was not available to make a proper comparison. However, the answers to these questions strengthen the understanding of the incidence of anomalies in dentition while establishing a precedent for invasive populations.

## 2. Materials and Methods

### 2.1. Study Area

**Figure 1:**
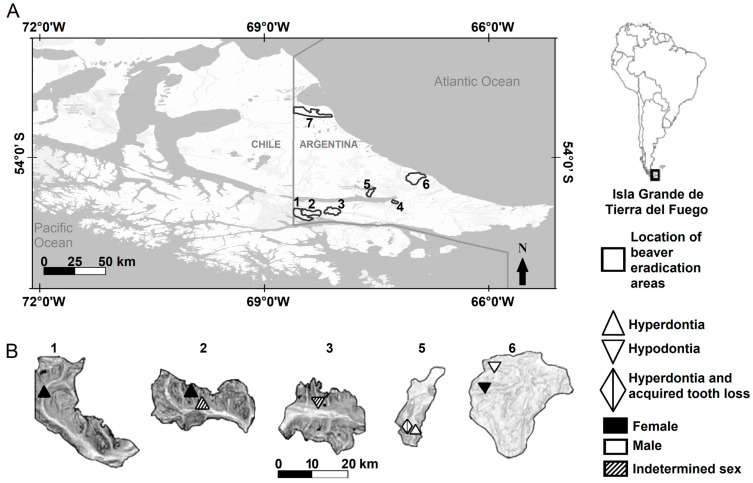
Locations of beaver eradication areas in the Isla Grande de Tierra del Fuego, Argentina (**A**), and locations of beavers with hyperdontia and hypodontia in five of seven areas (**B**). The scale refers to the magnified areas. Areas 1–4 were in the Mountain region (1 = Río Pipo, 2 = Arroyo Grande, 3 = Esmeralda, and 4 = Arroyo Indio), the areas 5–6 were in the Ecotone region (5 = Río Mimica and 6 = Arroyo Asturiana), and the area 7 was in the Steppe region (7 = Arroyo Gamma).

The beaver eradication areas are located in the Argentinian portion of Tierra del Fuego in three ecological regions: the Mountain region, the Ecotone region, and the Steppe region [41]. *Nothofagus* sp., Blume 1850, forest predominates in the Mountain and Ecotone regions. Vegetation in the Ecotone region, however, acquires a transitional aspect as latitude decreases. The Steppe region has a predominance of grasses (*Festuca* sp., Linnaeus 1753) and shrubs (*Chiliotrichum* sp., Cassini 1817, and *Berberis* sp., Linnaeus 1753) [41]. Four areas were in the southern Mountain (Río Pipo = 160 beavers [males = 74, females = 49, indeterminate = 37], Arroyo Grande = 137 beavers [males = 52, females = 65, indeterminate = 20], Esmeralda = 118 beavers [males = 63, females = 50, indeterminate = 5], and Arroyo Indio = 167 beavers [males = 75 females = 84, indeterminate = 8], Figure 1) and two areas in the Ecotone (Río Mimica = 119 beavers [males = 62, females = 49, indeterminate = 8] and Arroyo Asturiana = 90 beavers [males = 43, females = 41, indeterminate = 6], Figure 1). The Mountain and Ecotone are considered here as forested environments. One area was located in the northern part of the Steppe (Arroyo Gamma = 151 beavers [males = 70, females = 72, indeterminate = 9], Figure 1). In addition, 28 beavers were removed because they could not be assigned correctly to an area due to poorly preserved identification tags; the sex of these animals was recorded as indeterminate.

### 2.2. Beaver Eradication and Specimens Collection

All details on the beaver eradication program are available in previous studies [30]. Eradication followed the guidelines of the Agreement on International Humane Trapping Standards [42], as well as the Beaver Removal Manual of the Animal and Plan Health Inspection Service of EE. UU., and used information on the North Dakota Cooperative Fur Harvester Education Program. The government participated in approving the procedures, as well as the recruitment and training of hunters. In turn, the Global Environment Facility (financing agent, GCP/ARG/023/GEF) and the Food and Agriculture Organization of the United (administration agent) approved the trapping methodology [30]. A total of 1121 beavers were trapped, and heads were collected from 970 animals for laboratory analysis. The sex of beavers was determined by means of the presence of the baculum bone (439 males and 410 females [43]). The sex of 121 beavers was recorded as indeterminate due to post-trapping scavenging; however, these animals were included in the inspection to answer the questions proposed. To assess which age class showed the highest incidence of dentition anomalies, absence of teeth (by trauma or by disease), fractured teeth, and caries, the age at death of all collected beavers was estimated in years (yr) based on the dentition composition and the counting of incremental lines in the cementum portion of the molariform teeth [44]. To estimate age, the soft tissues were completely removed from the skulls by boiling them; the beaver skulls were deposited in the Centro Austral de Investigaciones Científicas (CADIC-CONICET, Ushuaia City).

### 2.3. Data Analysis

In order to assess the prevalence of hypodontia, hyperdontia, artifactual tooth loss, acquired tooth loss, caries, fractured teeth, and cranial abnormalities, a sample of 970 beaver skulls was examined from a total of 1121 animals removed. *Dental formula* of beaver is: Incisor tooth (I) = 1/1, Canine tooth = 0/0, Premolar tooth (Pm) = 1/1, and Molar teeth (M) = 3/3 = 20 [45]. Possession of any tooth that surpasses the typical number of deciduous or permanent teeth in any quadrant of dentition (left upper, left lower, right upper, and right lower) was considered hyperdontia. Non-traumatic/disease absence of one or more teeth—caused by congenital factors, as has been proposed in beavers [35]—was indicative of hypodontia. Instead, the artifactual tooth loss was determined when bone presented a remodeling condition, while the acquired tooth loss occurred by dental disease.

The incidence of fractured teeth was considered when one or more teeth presented a different shape and size and relatively healthy enamel state compared with the typical shape and size of non-fractured teeth. Teeth with caries were determined on the basis of poor enamel preservation with respect to teeth with a healthy dentition state. On the other hand, there are several malocclusion classification systems developed for human dentition [26] applied even in wild animals under the use of sedation and small sample size (e.g., *Phascolarctos cinereus*, Goldfuss 1817 [27]). The boiling process necessary to remove the soft tissue skulls and posterior dental extraction to age estimation made it impossible to apply a malocclusion classification system to the beavers assessed. Therefore, whether a skull showed improper tooth wear (a sign of an abnormal occlusion) was then recorded as malocclusion compared with skulls with adequate tooth wear. The malocclusion was classified according to the observed condition of dentition as malocclusion associated with fractured teeth, artifactual tooth loss, acquired tooth loss, or caries.

To assess whether the incidence (calculated as a percentage) of dentition anomalies, artifactual tooth loss, acquired tooth loss, fractured teeth, and caries vary across different invaded environments, beaver skulls were classified according to ecological region (Mountain, Ecotone, and Steppe). This association (even in those anomalies of congenital origin, such as hypodontia and hyperdontia) provides relevant information about anomaly effects on population parameters, for example, on lifespan. Because the number of areas by environment is unbalanced, with the Steppe being the region with the lowest number of eradicated areas (1), a statistical analysis was not performed. The percentage of anomalies mentioned was also calculated according to their incidence on the left or right lower quadrant, as well as on the left or right upper quadrant. To analyze whether the prevalence of hypodontia, hyperdontia, artifactual tooth loss, acquired tooth loss, fractured teeth, caries, and cranial abnormalities were equally represented in both sexes, a chi-squared test (χ^2^, *p*-value < 0.05) was applied using the InfoStat software version 2014p [46]. A similar analysis was performed to analyze whether prevalences were mainly represented in younger animals (beavers that survived up to 1 year-old) than in sub-adults (2 year-old) and adult animals (3 year-old onward). For this purpose, individuals with dentition anomalies or pathologies were grouped into a single category; equally, individuals with malocclusion or cranial abnormalities were grouped to reach test assumptions [47]. In turn, the sex ratio of animals with the same anomaly or condition of dentition or cranial was calculated and used to indicate a trend according to sex.

## 3. Results 

**Figure 2:**
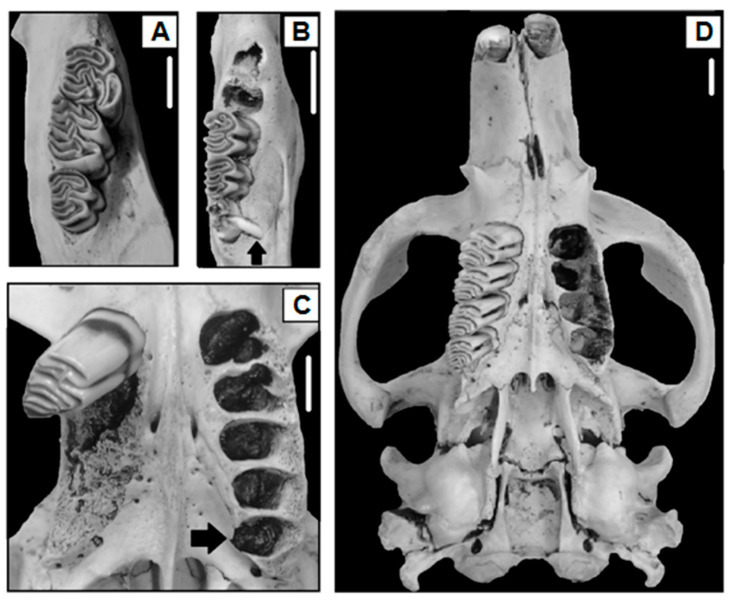
Examples of hypodontia ((**A**), right lower quadrant), hyperdontia ((**B**), right lower quadrant); hyperdontia and acquired tooth loss in the same animal ((**C**), left and right upper quadrants, respectively), and buccal curvature denoted by a marked intermaxillary suture deviation of the upper left arch ((**D**), cranial) in *C. canadensis*, Isla Grande de Tierra del Fuego, Argentina. Scales represent 1 cm. The lacking molar teeth in (**C**,**D**) were extracted for age estimation.

Of the 970 beaver skulls analyzed (sex ratio 1.07:1, male:female), hypodontia occurred in four beavers (Figure 2A), while hyperdontia was recorded in four beavers (Table 1 and Table 2, Figure 2B). One beaver that showed hyperdontia also presented acquired tooth loss (Table 1 and Table 2, Figure 2C). Three beavers showed artifactual tooth loss, and six presented acquired tooth loss (Table 1 and Table 2). The presence of fractured teeth by physical trauma was recorded in four beavers of the skulls analyzed, and three showed caries (Table 1 and Table 3). Nine beaver skulls presented a cranial abnormality characterized by a buccal curvature (deformed rostrum, Table 1 and Table 4, Figure 2D). On the other hand, five beavers presented an improper tooth wear “malocclusion” (Table 1), for which one case was related to fractured teeth (20%), two to caries (40%), and two cases were not related to an observed condition of the dentition “unknown” (40%, Table 5). Overall, the prevalence of anomalies and pathologies of the dentition and cranial did not differ between sexes (χ^2^ = 2.00, *p*-value = 0.15), nor between younger, sub-adult, and adult individuals (χ^2^ = 0.59, *p*-value = 0.74). However, some trends were observed according to the sex ratio, as presented below.

### 3.1. Prevalences According to Ecological Region

The dentition anomalies were located in five of the seven areas (Table 2, Figure 1). No hypodontia or hyperdontia was recorded in the Steppe (area of Arroyo Gamma). The beaver that showed hyperdontia and acquired tooth loss was collected from the Ecotone (Río Mimica, Table 2, Figure 2C). The beavers with conditions of tooth losses were recorded from forested areas (Table 2). Beavers from the Steppe (Arroyo Gamma) showed no fractured teeth or caries. On the other hand, the individuals with buccal curvature were recorded both in the Steppe (Arroyo Gamma) and in the forested areas in the Mountain (Table 4). Incidence of malocclusion was recorded at both Steppe (20%) and in the forested areas in the Mountain (80%). Only at Río Mimica, in the Ecotone, improper tooth wear was not found.

### 3.2. Prevalences According to Quadrants of the Dentition

The upper quadrants of dentition showed the lowest incidence of dentition anomalies (12.5%) and only featured hypodontia cases (Table 2). Hypodontia was recorded mainly in the left lower quadrant (75%), the third molar tooth being the most common lacked tooth (75%, Table 2). An adult male from Río Mimica showed hyperdontia and acquired tooth loss, both located on the left and right upper quadrants. Artifactual tooth loss and acquired tooth loss mainly affected the left upper quadrant (Table 2). Over half of all artifactual tooth losses (66.6%) occurred in premolar teeth (Table 2), while acquired tooth losses were related to molar teeth, mainly M3 (Table 2). The third molar tooth was most frequently fractured (75% of occurrences), followed by the premolar teeth (25%, Table 3). The frequency of fractured teeth was higher in the right lower quadrant and in the right upper quadrant than in the left quadrants (Table 3). Instead, caries occurred mainly in the left upper quadrant (Table 3). The lowest incidence of caries occurred in the first and second molar tooth (33.3% of the total of beavers with caries, Table 3) and was recorded in the same beaver. Malocclusion and buccal curvature mainly occurred in the right upper quadrant (80%).

### 3.3. Prevalences According to Sex

Hyperdontia was skewed toward females (0.50:1, male:female), and the sex ratio of beavers with hypodontia was 1:0.50 (male:female, Table 2). On the other hand, half of the hyperdontia cases occurred in females (50%), and they were more common in the right lower quadrant (75%), presenting an additional premolar tooth or molar tooth (M4, Table 2). The highest incidence of artifactual tooth loss and acquired tooth loss mainly affected females (sex ratio 0.6:1, male:female, Table 2). Females presented a higher incidence of fractured teeth in relation to males (0.3:1, male:female, Table 3); a similar sex-biased incidence was observed in animals with caries (0.5:1, male:female, Table 3). The buccal curvature and malocclusion mainly affected the females (sex ratio 0.2:1, male:female, Table 4 and Table 5).

### 3.4. Prevalences According to Age

Adult beavers (from 3 year-old onwards) accounted for a substantial number of cases of hypodontia or hyperdontia in contrast to earlier age classes (Table 2). The cases of artifactual tooth loss and acquired tooth loss were recorded in adult animals (89%, Table 2). Most fractured teeth and caries occurred in adult beavers (Table 3). Fifty-five percent of the buccal curvature was identified in beavers in their first years of life, and, in general, skulls showed a left-directed buccal curvature (Table 4), without any difference by sex (sex ratio 1:1, male/female). Adult beavers showed the highest malocclusion incidence (60%, Table 4). The cases of malocclusion that were not related to an observed condition of the dentition “unknown” were young beavers (40%, Table 5).

### 3.5. Malocclusion and Cranial Abnormalities

Malocclusion produced by fractured teeth and by caries affected only incisor teeth (Table 5). Moreover, five beaver adults (0.51% of the total assessed skulls) presented severe tooth wear, probably associated with aging, affecting the premolar teeth, as well as the first and second molar teeth (Table 5).

## 4. Discussion

The prevalence of hypodontia, hyperdontia, artifactual and acquired tooth loss, fractured teeth, and caries was low in the eradicated areas. The hypodontia and hyperdontia mainly affected the lower quadrants of dentition (75% for both anomalies). The results indicate that these anomalies and pathologies are random with respect to sex and age class, although some trends were observed. Beaver females manifested mostly hyperdontia. Although the third molar tooth showed the highest incidence of dentition anomalies, absence of teeth (by trauma or by caries), fractured teeth, and caries (followed by premolar teeth), affecting all age classes (with a substantial representation of adults), these would not, a priori, compromise the lifespan of invasive beavers. A low number of skulls presented a left-directed buccal curvature. Malocclusion cases were probably related to fractured teeth or to caries. The results of this study were obtained from beavers collected to assess the feasibility of eradication, being the only study with age- and sex-specific data from a “population census”. Previous studies assessed dental anomalies in beavers (including *Castor fiber*, Linnaeus 1758) in other areas of distribution of the species using a smaller sample size [35,36,37,38] than in the present research. Although there is no similar sample of North American beaver skulls in their native range to compare to the results obtained in this study, the available background allows us to reinforce the results of this research. Nevertheless, comparisons of results between the present study and previous studies (with different sample sizes and specimen collection) should be made cautiously.

Some backgrounds suggest that the prevalence of dentition anomalies in beavers represents a recessive hereditary factor that has no selective meaning [35]. The proportions of hypodontia (0.41%, *n* = 4 animals) and of hyperdontia (0.41%, *n* = 4 animals) in the areas were lower than the proportion reported in a sample of 14 North American beavers (21.42%, *n* = 3 animals [36]). Other studies reported 15% of hypodontia in animals examined and 7% of hyperdontia [37,38]. The proportion of anomalies on *C. fiber* was also higher (3.17%, *n* = 8 animals of a sample of 252 beavers [35]) than in the studied areas. Despite their congenital origin, these dentition anomalies that cause a deviation from typical dentition patterns are not considered the degradation of a dysfunctional tooth [35].

### 4.1. Prevalences According to Ecological Region

The dentition anomalies, absence of teeth (by trauma or by caries), fractured teeth, and caries only occurred in the forested areas of the Mountain and the Ecotone. Although the tree species (e.g., *Populus* sp., Linnaeus 1753) with which beavers coevolved in North America are absent in Tierra del Fuego, the Fuegian forest environments “provide” some requirements (but does not necessarily constitute optimal habitats for the species) for beaver dentition health (e.g., substrate for gnawing and tooth wear). The absence of fractured teeth and caries in the Steppe, a region devoid of forest masses, was notable. The predominance of grasses and shrubs in the Steppe, which are part of the beaver’s diet (personal observation), could play an important role in the absence of dentition pathologies and fractured teeth compared with the dental health of beavers in forested areas. The highest incidence of fractured teeth and of caries in the third molar tooth could be associated with its delayed eruption, which, by physical stress, can also affect antagonist pieces [35]. This could explain the highest predisposition of damage to the third molar tooth in all forested areas. A similar reflection could be postulated for premolar teeth, which, due to their deciduous nature, likely increase damage to this tooth when the deciduous premolar tooth is replaced by a permanent premolar tooth. On the other hand, the incidence of buccal curvature or malocclusion cases did not show any predisposition according to the environment, which suggests that their incidence could be random and not associated with the habitat from where the beavers were eradicated.

### 4.2. Prevalences According to Quadrants of the Dentition

Beavers are known to develop dental anomalies in the lower quadrants of the dentition [35,48]. Invasive beavers showed this pattern; surprisingly, hyperdontia and acquired tooth loss were recorded in the upper quadrant of the dentition of the same beaver (male). Another medium-sized rodent, *Myocastor coypus*, Molina 1782, however, showed dentition anomalies in the upper quadrants of dentition [7]. On the other hand, in small rodents, these anomalies can be present on either the upper or lower quadrants of dentition [12,49,50]. In beavers (*C. fiber*), some studies indicated a high prevalence of hypodontia and hyperdontia in the left lower quadrant (62.50% of a total of 22 individuals with anomalies [35]). Although invasive beavers also showed a high proportion of these anomalies in the lower quadrants, it was noted that hypodontia tended to develop mainly on the left lower quadrant (75%) and hyperdontia on the right lower quadrant of dentition (75%). 

Due to their vestigial nature, the teeth occupying a terminal position in the dental formula of mammals tend to be the most frequently absent [49]. The incidence of hypodontia in beavers showed that the third molar tooth was the tooth with the highest absence [33,48]. In the eradicated areas, hypodontia was characterized by the absence of a third molar tooth (75%). Hypodontia in *C. fiber* has been related to a recessive genetic composition, with discounted environmental factors as drivers of dental anomalies [35]. Due to the displacement capacity of the mandibles, beavers with hypodontia may realize adequate wear of antagonist pieces [35]; it is likely that this same adequate wear capacity also occurs in beavers with extra teeth in Tierra del Fuego, based on the estimated longevity of up to 9 years old in beavers with hyperdontia. On the other hand, hyperdontia in beavers has been recorded for premolar teeth [36]. Invasive beavers showed hyperdontia by means of an extra molar tooth and, to a lesser extent, premolar teeth; this has not been reported in previous studies.

The incidence of artifactual tooth loss, acquired tooth loss, fractured teeth, and caries in invasive beavers were low and occurred mostly in the lower quadrants of dentition. Wild animals typically show a low incidence of caries compared with captured individuals [3]. Several positional anomalies have been recorded, mainly in the third molar tooth of *C. fiber*, which was associated with ecologic-anatomical factors [35]. Tooth anomalies can cause starvation and, consequently, mortality in *C. fiber* in up to 0.9% of individuals (10 animals out of a sample of 1137 beavers [31]). Carious teeth and signs of parodontosis have also been reported in translocated beavers [38]. 

### 4.3. Prevalences According to Sex

Background information indicates that male beavers tend to manifest more dentition anomalies (62.50%) than females (25.0%) [35]. Although the anomalies and pathologies of the dentition or cranial significantly did not prevail over one sex, the sex ratio for those beavers with hyperdontia was skewed toward females, while hypodontia occurred mainly in males. The sex ratio of beavers with the absence of teeth (by trauma or by caries), fractured teeth, and caries (even malocclusion) showed a female bias, but its incidence would not represent a condition that affects the life history traits of the beavers. 

### 4.4. Prevalences According to Age

Contrary to expected, the incidence of dentition anomalies, absence of teeth (by trauma or by caries), fractured teeth, and caries, as well as cranial abnormalities, were recorded in both younger and older beavers. Fractured teeth and caries were present in all age classes, although mainly affecting adults (>3 year-old). These anomalies, as well as the cases of absence, fractured teeth, and caries, did not appear to be detrimental to beaver survival; this reflection was also proposed for other orders of mammals (e.g., *Lutra canadensis*, Schreber 1777 [9], *Lontra longicaudalis*, Olfers 1818 [51]). For example, some studies indicate that skeletal diseases and tooth defects are more frequent in older individuals [52]. Although cases of malocclusion and buccal curvature persisted with age, the highest proportion of buccal curvatures in young beavers could be related to traumatic events that cause high mortality rates in the first years of beavers’ lives [38]; this could explain the lowest incidence of buccal curvatures in adult animals.

### 4.5. Malocclusion and Cranial Abnormalities

Anecdotal cases of malocclusion in beavers have been reported in the incisor teeth of *C. canadensis* [53] and *C. fiber* [54]. In the invasive beavers, malocclusion was probably related to fractured teeth and caries and was denoted mainly by improper tooth wear (affecting the incisor teeth, premolar teeth, and first and second molar teeth) in the right upper quadrant of dentition. On the other hand, buccal curvature was a cranial abnormality that had not been reported previously in beavers. In medium-size rodents, such as *M. coypus*, cranial curvature has been associated with trauma [55]; some skulls of *O. zibethicus* in Tierra del Fuego presented certain asymmetrical tendencies [30].

The limitations of the study are as follows. Despite the fact that a representative sample of the invasive beaver population was analyzed, some limitations must be clarified. The potential impact of the lack of sex identification in some beavers in the evaluation of the prevalence of dentition anomalies must be considered; thus, this factor may have likely biased some estimations. The lack of a generalized analysis to measure the effect of the environment (e.g., the effect of grass feeding in the Steppe) on beaver dentition health is recognized; thus, it is suggested to develop a line of research to evaluate this topic. The difference in the number of areas from the different environments could affect the perception of the “absence” of the dentition conditions in the Steppe, which has the smallest sample size. In addition, it is recognized that a representative sample of beavers in their native range was not available to make a proper comparison. However, the age- and sex-specific results on the dentition anomalies and conditions in invasive beavers in Tierra del Fuego represent an unprecedented event since the population was collected totally at the local level.

## 5. Conclusions

The dentition anomalies and pathologies recorded in the beavers examined in this study occurred in Fuegian forest environments. It is highlighted the finding of dentition anomalies in the upper quadrants of dentition. These invaded forest environments could act as selective pressures that drives adaptive changes over short periods of time in beaver dentition. Despite the fact that the invasive beavers presented several dentition conditions, the low incidence suggests that their effect on the health status of the populations could be negligible. These results also call for reflection on why it is so difficult to find transitional species in the evolutionary process in the fossil record. Regarding non-congenital absence of teeth, their incidence could be related to physical trauma and caries, as well as their delayed eruption. The buccal curvature of skulls is reported for the first time in the species, and their higher incidence in younger beavers, compared with older animals, could be considered as a new mortality factor that affects the early life of the beaver. The fractured teeth and caries are predisposing factors for malocclusion of the incisor teeth, while inadequate tooth wear of premolars and molars teeth could be associated with aging. This study hopes to be a contribution to the understanding of dentition anomalies and cranial abnormalities in an invasive mammal species.

## Figures and Tables

**Table 1:** Frequency and proportion of specimens of *C. canadensis* with any dentition anomaly, pathology or cranial condition in invaded areas from Isla Grande de Tierra del Fuego, Argentina.

Dentition Anomalies and Pathologies and Cranial Condition	Frequency of Specimens	Proportion of Specimens with Respect to the Total Sample Analyzed (*n* = 970)
Hypodontia	4	0.41
Hyperdontia	4	0.41
Artifactual tooth loss	3	0.30
Acquired tooth loss	6	0.61
Fractured teeth	4	0.41
Caries	3	0.31
Buccal curvature	9	0.93
Malocclusion	5	0.51

**Table 2:** Hyperdontia, hypodontia, and fractured teeth by physical trauma in areas from Isla Grande de Tierra del Fuego, Argentina. Premolar tooth = Pm. Molar tooth (number of tooth) = M1, 2, 3 or 4. Age in years. / = indicates that the anomaly occurred in different quadrants of dentition.

Area	Age	Region	Sex	Quadrant of Dentition	Hyperdontia	Hypodontia	Artifactual Tooth Loss	Acquired Tooth Loss
Río Pipo	9	Mountain	Female	Right lower	Pm			
Esmeralda	10	Mountain	Indeterminate	Left/and right lower		M3/M3		
Arroyo Grande	1	Mountain	Female	Right lower	M4			
Arroyo Grande	0.6	Mountain	Indeterminate	Right lower	M4			
Río Mimica	8	Ecotone	Male	Left/and right upper	M4			M1,2,3
Río Mimica	2	Ecotone	Male	Right lower		M3		
Arroyo Asturiana	2	Ecotone	Female	Left lower		M3		
Arroyo Asturiana	5	Ecotone	Male	Left lower		Pm		
Esmeralda	12	Mountain	Female	Left upper			Pm, M1,2,3	
Arroyo Grande	2	Mountain	Female	Left lower				Pm
Río Pipo	4	Mountain	Female	Left upper			M3	
Río Mimica	4	Ecotone	Male	Left lower				M3
Arroyo Grande	8	Mountain	Indeterminate	Right lower			Pm	
Arroyo Grande	12	Mountain	Male	Left lower				M3
Arroyo Indio	4	Mountain	Female	Left lower				M3
Arroyo Indio	3	Mountain	Female	Right upper				Pm

**Table 3:** Fractured teeth by physical trauma and caries in *C. canadensis* removed in areas from Isla Grande de Tierra del Fuego, Argentina. Premolar tooth = Pm. Molar tooth (number of tooth) = M1, 2, or 3. Age in years. / = indicates that fractured teeth or caries were recorded in different quadrants of the dentition.

Area	Age	Region	Sex	Quadrant of Dentition	Fractured Teeth	Caries
Río Pipo	6	Mountain	Female	Left upper	M3	
Río Pipo	10	Mountain	Female	Left upper		M3
Río Pipo	9	Mountain	Male	Left upper		M1,2,3
Río Pipo	6	Mountain	Male	Right lower	M3	
Río Mimica	10	Ecotone	Female	Right upper		M3
Esmeralda	2	Mountain	Female	Right lower	Pm	
Esmeralda	13	Mountain	Female	Right upper/Right lower	M3/Pm	

**Table 4:** Buccal curvature of *C. canadensis* in areas from Isla Grande de Tierra del Fuego, Argentina. Age in years.

Area	Age	Region	Sex	Direction of Buccal Curvature
Esmeralda	10	Mountain	Indeterminate	Left
Esmeralda	1	Mountain	Male	Left
Río Pipo	9	Mountain	Female	Left
Arroyo Indio	2	Mountain	Female	Right
Arroyo Indio	2	Mountain	Male	Right
Arroyo Grande	2	Mountain	Female	Left
Arroyo Gamma	5	Steppe	Female	Left
Arroyo Gamma	9	Steppe	Male	Left
Arroyo Gamma	2	Steppe	Male	Right

**Table 5:** Improper tooth wear in dentition of *C. canadensis* in areas from Isla Grande de Tierra del Fuego, Argentina. Incisor tooth = I, Premolar tooth = Pm, Molar tooth (number of tooth) = M1, 2, or 3. Age in years. / = indicates that improper tooth wear was recorded in different quadrants of the dentition.

Area	Age	Region	Sex	Quadrant of Dentition	Improper Tooth Wear Produced by			Severe Tooth Wear Associated with Aging
					Fractured Teeth	Caries	Unknown	
Arroyo Indio	13	Mountain	Female	Right upper/Left and right lower		I/I,I		
Arroyo Indio	11	Mountain	Male	Right upper/Left lower	I/I			
Arroyo Grande	13	Mountain	Female	Right upper		I		
Esmeralda	1	Mountain	Female	Right upper			Pm, M1,2	
Arroyo Gamma	2	Steppe	Female	Right upper			Pm, M1,2,3	
Arroyo Asturiana	5	Ecotone	Male	Left/and right lower				Pm/Pm
Río Pipo	11	Mountain	Male	Right lower				Pm, M2
Arroyo Gamma	9	Steppe	Male	Right upper				M1,2
Arroyo Indio	7	Mountain	Male	Left/and right lower				Pm/Pm
Arroyo Indio	7	Mountain	Female	Right lower				Pm

## Data Availability

No new data were created or analyzed in this study. Data sharing is not applicable to this article. Beaver skulls were deposited in the Centro Austral de Investigaciones Científicas (CADIC-CONICET, Ushuaia City).
